# Factors affecting the work competency and stability of family doctors in Shanghai: a tracking study

**DOI:** 10.1186/s12875-019-0988-6

**Published:** 2019-07-06

**Authors:** Shanshan Liu, Luan Wang, Tao Zhang, Chengjun Liu, Hong Liang, Yimin Zhang, Dongfeng Guo

**Affiliations:** 1 Pudong Institute for Health Development, Shanghai, China; 2 Shanghai Sixth People’s Hospital East Affiliated to Shanghai University of Medicine & Health Sciences, Shanghai, China; 3 Jinyang Community Health Service Center of Pudong New Area, Shanghai, China; 4 School of Social Development and Public Policy, Fudan University, Shanghai, China; 5 Eye and dental diseases prevention & treatment of Pudong new area, Shanghai, China; 6 Department of General Medicine, Gongli Hospital, the Second Military Medical University, Shanghai, China

**Keywords:** Family doctors, Competencies, Stability, Influential factors

## Abstract

**Background:**

Family doctors can fulfill the gatekeeper duty to protect the residents’ health, which depends on their work competency and stability. The study aimed to identify factors influencing work competency and stability among Shanghai family doctors.

**Methods:**

This study was a 2-year follow-up survey (2013/2016). A representative sample of 146 family doctors in Shanghai community health service centers was interviewed in 2013. The tracked sample (*n* = 142) was resurveyed in 2016. A 50-item questionnaire organized into four parts, i.e., general demographic characteristics, working conditions, cognition about family doctor services, and job satisfaction was issued to all family doctors. Models for factors influencing family doctors’ work competency and stability were then established. The collected data were analyzed using ordinal regression methods and descriptive, factor, and multiple-factor analyses.

**Results:**

The family doctors’ work competency model showed demographic characteristics (education level and job title), family doctors’ team, family doctors’ training, grasp of specific content regarding the family doctor system, and whether the family doctors’ ability was played and demonstrated were statistically significant at different levels (*P* < 0.05). The analysis of family doctors’ work stability showed that work competency (whether it was possible to provide residents with all contents specified in the contract service package, employment form, and support satisfaction) and work cognition (whether the daily work was meaningful and had value) had a significant impact on work stability (*P* < 0.05).

**Conclusion:**

Family doctors’ job satisfaction is a key factor affecting their work stability. Family doctors’ competency can also affect their job stability, and their work cognition may play a role in work competency and stability. This study provides evidence for strengthening the stability of the family doctor teams in Shanghai.

## Background

The number of patients with chronic diseases is increasing as aging population accelerates. Services provided by family doctors play an important and active role in the provision of community health care services, particularly in the rational use of health resources and improvement of community residents’ health status [1–3]. In 1997, the Decision of the Central Committee of the Communist Party of China and the State Council on Health Reform and Development first proposed the training of specialized general practitioners. In 2009, the Opinions of the Central Committee of the Communist Party of China and the State Council on Deepening the Reform of the Medical and Health System proposed the family doctor system as the working goal of community health services. In 2016, the State Council Medical Reform Office issued Opinions on Promoting Family Doctors’ Contracting Services, which marked the launch of the family doctor contracting service in China’s urban and rural primary health care service institutions. In 2018, Opinions of Regulations on Family Doctor Contracting Services Management was proposed to enhance the standardized management level of family doctors’ contracting services and promote the quality and efficiency of family doctors’ contracting services. China’s 13th Five-Year Plan clearly defines the key task of promoting family doctor contracting services under the premise of voluntarily registering residents to their clinical practices during the current 5-year period [4]. The construction of a family doctor system has passed through the theoretical systematic, and practical stages in China.

The family doctor contracting services, is based on the general practitioners and supported by the family doctor service team. By signing the contract, the general (clinical) doctors can establish a long-term and stable service relationship with the contracted families, to provide safe, convenient, effective, continuous, economical basic medical services and basic public health services for the contracted families and individuals. Shanghai was the first city to implement the family doctor contracting system in China as a basic component of Shanghai’s medical and health reform. In 2011, the family doctor contracting system was officially launched in Shanghai. In 2015, Shanghai launched a new round of comprehensive reform of the community health services by releasing the “1 + 1 + 1” medical institution contracting services (i.e., residents can voluntarily choose a family doctor from a community health service center, a secondary hospital, and a municipal hospital to sign their contracting services). As of 2018, 6.66 million people became contracted residents in Shanghai’s “1 + 1 + 1” medical institution portfolio, of whom 30% were permanent residents. On May 19, 2019, the 9th “World Family Doctor Day” carried out a series of publicity activities with the theme of “working with family doctors and building a healthy life together” to create a good social atmosphere that supporting contracting services and family doctors.

Family doctors are oriented towards their community’s needs and emphasize their practical availability to their community. In addition, family doctor contracting services are people-oriented and can meet the population’s health needs by providing continuous, comprehensive, and long-term services for families and communities. Various factors affect family doctors’ provision of services as contracted service providers, including: whether they are capable of contracting services, whether the services are attractive to the community residents, and whether there is any incentive policy for contracting services. Therefore, a family doctor’s work competency is crucial for integrating an individual’s knowledge, skills, judgments, and attitudes. Competency was first proposed by David McClelland in 1973 [5] and refers to the deep-seated characteristics of individuals who can distinguish between those who have outstanding achievements in a job and ordinary people, Competencies could include motivations, traits, self-images, attitudes or values, and any knowledge, cognitive, or behavioral skills in a fields that can be reliably measured or counted. Thus, competency can be used to significantly distinguish between good and general performance. The competency model is a competency structure that combines the outstanding competency requirements of specific positions. The foreign general practitioner competency model has been well established, such as the World Organization of National Colleges, Academies and Academic Association of General Practitioners/Family Physicians (WONCA) proposes a general practitioner’s core competency model that includes the six core competencies: i.e., primary care management, people-centred care), specific problem solving skills, comprehensive approaches, community orientation, and holistic modelling. Domestic research on the competence of general practitioners in community health service institutions has been performed, mostly around professional knowledge, interpersonal relationships, professional ethics, communication skills and communication skills, and work enthusiasm [6–9]. Compared with studies in foreign countries, domestic competency studies were generally lacking in the current state of theoretical research.

Therefore, family doctors not only have the ability to provide community health services, but also need to be able to provide continuous service capabilities and high quality of service, which depends on the stability and enthusiasm of the general practitioner’s team. Studies have identified an “active” flow of doctors in primary health care institutions is “active” [10–12], which directly affects the provision of family doctors’ contracting services and the sustainable development of community health service centers.

However, the expansion of the family doctor contracting service exposed some problems in the health-care system. For example, general practitioners had some cognitive differences regarding service provision and were not satisfied with their payments, and the residents’ registration rate of residents was not high [13–15]. Based on the above, this study drew on the domestic and international general practitioners’ competency and stability model to identify factors affecting the work competency and stability of family doctors in community health service centers. Changes in influential factors were also tracked to provide a realistic basis for understanding how family doctors can fulfill their gatekeeper duty to protect their residents’ health as well as deepen the reform of community health-care services.

## Methods

### Literature review

The terms “community health service” “family doctor” “general practitioner” “competence” and “stability” were used as key search words. We searched the China Knowledge Network, Wanfang Data, PubMed, and Elsevier databases. More than 200 relevant references were systematically searched to determine the research status and trends for family doctors’ work abilities and summarize the relevant survey indicators for the research group.

### Expert advice

Eleven experts from universities, academic research institutions, administrative departments, and community health service centers were identified, and an expert centralized demonstration meeting was held. In combination with community health service reform and family doctor system construction, the research group designed a questionnaire was formulated by the research group. Based on the consultation results, this questionnaire was modified to enhance the importance, accessibility, and operability of the relevant survey indicators.

### Questionnaire survey

This study involved a 2-year follow-up survey (2013/2016). The participants were all family doctors who passed their assessment and certification and provided contracted services in the community health service centers of Changning District and thus were eligible for enrollment in this study.

Changning District has been exploring family doctor services since 1997. So far, the family doctors system has experienced four versions of the upgrade. In the 3.0 version of family doctors system between 2010 and 2015 year, the family doctors were the main contracting body to providing contracting services, with the goal of graded medical treatment and health management. Furthermore, in the 4.0 version of family doctors system (since 2016 year), the contracting object expanded from the key residents to all residents, with the goal of improving service platform and capabilities. So we chose Changning District as the investigation site. This district was the pilot area for the family doctor system reform in Shanghai; thus, these family doctors better represented the reform results. The research team had a good working relationship with the Changning District community health centers; therefore, it was convenient to use these centers to facilitate the investigation and research.

### Sample size and method

The research team obtained full coverage of the general practitioners who worked as family doctors in Changning District. The first investigation was conducted in December 2013. The follow-up survey of family doctors was conducted in August 2016. In total, 154 respondents met the requirements in the first-phase survey, and 152 completed questionnaires were valid. There were 143 respondents in the second-phase follow-up survey, with 142 valid questionnaires.

Based on the relevant domestic and foreign literature, we developed the “*Family Doctor Questionnaire for Shanghai Community Family Doctors and Graded Medical Treatment System Assessment*” as the survey instrument. The questionnaire was divided into four parts involving 50 items, such as basic personal information (7 items), work competency (15 items), family doctors’ perceptions (17 items), and job satisfaction (11 items) respectively.

A member of the research group was responsible for distributing and collecting the questionnaire, which was sent to community health center directors and used an anonymous self-reporting method. After eliminating invalid questionnaires, the data were entered using EpiData Software (v. 3.1; EpiData Software, Redwood City, CA, USA) following the parallel double-entry method.

Two methods for model construction were used in this study. The first model examined factors influencing family doctors’ work competency. The dependent variable was “*whether it was possible to provide residents with all content specified in the contract service package*.” Independent variables were demographic characteristics, family doctors’ qualification and job training, family doctors’ team configuration and relationships, and family doctors’ cognition including 15 items. The second model explored factors influencing family doctors’ work stability. The dependent variable was “*if you have the opportunity, whether you will accept the work of second- and third-level hospitals*”. The independent variables were demographic characteristics, family doctors’ cognition, work competency, and job satisfaction including 13 items.

### Statistical analysis

The data were input using EpiData 3.1 software (v. 3.1). Statistical analyses were performed using SPSS (v. 19; IBM SPSS, Armonk, NY, USA) and SAS software (v. 9.2; SAS Institute, Cary, NC, USA). The analytical methods included ordinal regression methods, and descriptive (mean and standard deviation, or number and percentage), factor, and multiple-factor (repeated measurement model and others) analyses.

## Results

### Analysis of respondents’ demographic characteristics

In 2013, 146 family doctors were included in the survey; 64.38% were women, and 44.14% were aged 30–39 years. The most common education level was a bachelor’s degree, accounting for 73.97%. Most respondents (73.61%) held intermediate titles, and 47.18% had worked for ≥4 years. In 2016, 142 family doctors were included in the survey: 68.31% were women and 52.11% were aged 30–39 years. The most common education level was a bachelor’s degree(77.46%). Most respondents (83.57%) held intermediate titles, and 86.33% had worked for more than 4 years (Table 1).

**Table 1:** Demographic characteristics of participating family doctors

Variable	2013		2016	
	Number (N)	Percentage (%)	Number (N)	Percentage (%)
Gender				
Male	52	35.62	45	31.69
Female	94	64.38	97	68.31
Age				
0~29	23	15.86	5	3.52
30~39	64	44.14	74	52.11
40~49	30	20.69	33	23.24
≥ 50	28	19.31	30	21.13
Education				
High school and below	4	2.74	0	0.00
College degree	24	16.44	19	13.38
Bachelor’s degree	108	73.97	110	77.46
Master’s degree and above	10	6.85	13	9.15
Title				
No title	1	0.69	0	0.00
Primary title	34	23.61	9	6.43
Intermediate title	106	73.61	117	83.57
Senior title	3	2.08	14	10.00
Time as a family doctor (year)				
0~2 years	24	16.90	7	5.04
2~4 years	51	35.92	12	8.63
More than 4 years	67	47.18	120	86.33

### Analysis of factors influencing family doctors’ work competency

The model of influential factors impacting family doctors’ work competency was based on “whether it was possible to provide residents with all content specified in the contract service package” as the dependent variable (the independent variables were: demographic characteristics, family doctors’ qualification and job training, family doctors’ team configuration and relationships, and family doctors’ cognition). In 2013, demographic characteristics (education, job title), family doctors’ team, family doctors’ training, grasp of specific contents of the family doctor system, and whether the family doctors’ ability was played and demonstrated were statistically significant at different levels (*P* < 0.05). In 2016, family doctors’ training and whether the family doctors’ ability was played and demonstrated were statistically significant at different levels (*P* < 0.05) (Table 2).

**Table 2:** Analysis of factors influencing family doctors’ work competency

Variable		2013					2016				
		Estimate	Wald	*P*	95%CI		Estimate	Wald	*P*	95%CI	
					Lower Bound	Upper Bound				Lower Bound	Upper Bound
Age		−0.033	0.096	0.757	−0.240	0.175	−0.029	0.075	0.785	−0.239	0.180
Time of working(year)	Engaged in clinical work	−0.005	0.004	0.950	−0.158	0.149	0.044	0.133	0.716	−0.192	0.279
	Engaged in community health service	−0.072	0.960	0.327	−0.215	0.072	−0.051	0.408	0.523	−0.208	0.106
	Working as a family doctor	−0.198	2.134	0.144	−0.464	0.068	−0.118	0.697	0.404	−0.395	0.159
	Working in community health service center	0.062	0.605	0.436	−0.095	0.219	0.038	0.219	0.640	−0.122	0.198
Gender	Male	−1.044	1.702	0.192	−2.611	0.524	0.591	0.464	0.496	−1.109	2.290
	Female	0	.	.	.	.	0	.	.	.	.
Education	High school and below	8.485	1.728	0.189	−4.166	21.135	–	–	–	–	–
	College degree	7.331	9.958	0.002	2.778	11.885	2.746	1.450	0.228	−1.723	7.215
	Bachelor’s degree	5.078	9.212	0.002	1.799	8.357	1.716	1.287	0.257	−1.249	4.681
	Master’s degree and above	0	.	.	.	.	0	.	.	.	.
Title	Primary title	3.747	2.855	0.091	−0.599	8.092	3.265	2.096	0.148	−1.155	7.685
	Intermediate title	5.377	6.610	0.010	1.278	9.476	2.299	1.642	0.200	−1.217	5.816
	Senior title	0	.	.	.	.	0	.	.	.	.
Physician classification	General practitioner	0.252	0.017	0.895	−3.479	3.982	−0.725	0.488	0.485	−2.762	1.311
	Clinician practitioner	−0.152	0.003	0.957	−5.640	5.337	−1.284	0.351	0.554	−5.532	2.965
	Chinese medicine practitioner	0.507	0.058	0.810	−3.622	4.636	0	.	.	.	.
	Other	0	.	.	.	.	–	–	–	–	–
Employment forms	Officially edited	1.891	0.292	0.589	−4.970	8.752	3.750	3.226	0.072	−0.342	7.842
	Labor dispatch system	3.168	0.769	0.381	−3.913	10.249	1.375	0.181	0.671	−4.966	7.715
	Temporary contract system	–	–	–	–	–	3.713	0.887	0.346	−4.014	11.440
	Other	0	.	.	.	.	0	.	.	.	.
General practitioner qualification	Yea	−1.100	0.559	0.455	−3.984	1.784	−0.260	0.016	0.900	−4.318	3.797
	No	0	.	.	.	.	0	.	.	.	.
General practitioner standardized training	Yea	−1.306	2.971	0.085	−2.792	0.179	−0.823	0.930	0.335	−2.497	0.850
	No	0	.	.	.	.	0	.	.	.	.
Team configuration	Reasonable	−3.211	6.642	0.010	−5.653	−0.769	−0.555	0.239	0.625	−2.782	1.672
	Unreasonable	0.404	0.243	0.622	−1.201	2.009	−1.325	1.623	0.203	−3.363	0.713
	Uncertain	0	.	.	.	.	0	.	.	.	.
Relationship with the team	Parallel position	1.727	2.145	0.143	−0.584	4.039	0.132	0.008	0.930	−2.831	3.095
	Core position	0.041	0.002	0.968	−1.946	2.028	0.136	0.008	0.927	−2.795	3.068
	Passive status	−1.105	1.019	0.313	−3.249	1.040	0.626	0.070	0.792	−4.020	5.272
	Complex relationship	0	.	.	.	.	0	.	.	.	.
Job training	Necessary	−4.164	7.052	0.008	−7.237	−1.091	2.092	1.519	0.218	−1.235	5.419
	Occasionally required	−4.133	7.884	0.005	−7.018	−1.248	3.172	3.863	0.049	0.009	6.336
	Not required	−5.436	8.824	0.003	−9.023	−1.849	0	.	.	.	.
	Never required	0	.	.	.	.	–	–	–	–	–
Grasping the specific content of the family doctor system	Very clear	−4.944	2.265	0.132	−11.383	1.494	−2.495	0.809	0.368	−7.930	2.940
	Clearer	−6.715	4.507	0.034	−12.914	−0.516	−1.752	0.518	0.472	−6.526	3.021
	General	−5.425	3.223	0.073	−11.348	0.497	0	.	.	.	.
	Not sure	0	.	.	.	.	–	–	–	–	–
Clear about the job responsibilities	Very clear	3.296	0.619	0.432	−4.917	11.509	0.942	0.101	0.751	− 4.873	6.757
	Clearer	5.618	1.825	0.177	−2.532	13.769	2.720	0.855	0.355	−3.046	8.486
	General	1.399	0.135	0.713	−6.066	8.864	0	.	.	.	.
	Not sure	0	.	.	.	.	–	–	–	–	–
Ability to play and demonstrate as a family doctor	Fully play	24.394	176.421	0.000	20.794	27.993	−4.766	5.320	0.021	−8.816	−0.716
	Play	21.458	364.736	0.000	19.255	23.660	− 0.613	0.433	0.510	−2.436	1.211
	General	22.066	450.147	0.000	20.028	24.105	0	.	.	.	.
	Difficult to play	23.176	.	.	23.176	23.176	–	–	–	–	–
	Not able to play	0	.	.	.	.	–	–	–	–	–
Log pseudo likelihood		95.414					77.315				
Chi-square value (*P* value)		68.534 (0.001)					50.130 (0.012)				
Pseudo R^2^		0.479					0.391				

### Factor analysis of family doctors’ job satisfaction

This analysis classified items for family doctors’ job satisfaction items and extracted three common factors according to the standard of common factor greater than 1. The first factor was labeled “collaborative environment satisfaction” and included satisfaction with equipment condition, office conditions, medical technology system support, organizational management, administrative logistics support, title promotion, informationization, and superior hospital collaborative diagnosis and treatment. The second factor was labeled “income satisfaction” and included satisfaction with welfare, job value, income level, and income and dedication fairness. The third factor was labeled “support satisfaction” and included satisfaction with general practitioner team support, relationships with colleagues, community (neighborhood) support, and leadership quality and management ability (Table 3).

**Table 3:** Factor analysis of family doctors’ job satisfaction (rotation component matrix)

Item	Common factors		
	1	2	3
Equipment condition satisfaction	0.911	0.066	0.019
Office conditions satisfaction	0.839	−0.036	0.007
Medical technology system support satisfaction	0.768	0.099	0.321
Organizational management satisfaction	0.723	0.153	0.493
Administrative logistics support satisfaction	0.712	0.081	0.474
Title promotion satisfaction	0.609	0.091	0.589
Informationization satisfaction	0.588	0.262	0.070
Superior hospital collaborative diagnosis and treatment satisfaction	0.322	0.159	0.269
Welfare satisfaction	0.146	0.919	0.045
Job value satisfaction	0.067	0.910	0.037
Income level satisfaction	0.087	0.819	−0.056
Income and dedication fairness satisfaction	0.101	0.804	0.080
General practitioner team support satisfaction	0.114	−0.027	0.731
Colleagues relationship satisfaction	0.258	0.022	0.711
Community (neighborhood) support satisfaction	−0.052	− 0.010	0.711
Leadership quality and management ability satisfaction	0.424	0.072	0.654

### Analysis of factors influencing family doctors’ work stability

The model of factors affecting family doctors’ work stability was based on “if you have the opportunity, would you will accept the work of second- and third-level hospitals” as the dependent variable. The independent variables were demographic characteristics, family doctors’ cognition and competency, and family doctors’ job satisfaction. The 2013 analysis showed that employment form and satisfaction with support had a significant effects on work stability (*P* < 0.05). In 2016, “whether it was possible to provide residents with all contents specified in the contract service package” and work cognition (“whether daily work was meaningful and had value”) significantly impacted work stability (*P* < 0.05). (Table 4).

**Table 4:** Analysis of factors influencing family doctors’ work stability

Variable		2013					2016				
		Estimate	Wald	*P*	95% CI		Estimate	Wald	*P*	95% CI	
					Lower Bound	Upper Bound				Lower Bound	Upper Bound
Age		−0.036	0.375	0.540	−0.153	0.080	−0.019	0.058	0.810	−0.173	0.136
Gender	Male	0.086	0.032	0.858	−0.856	1.028	−0.094	0.034	0.854	−1.092	0.904
	Female	0	.	.	.	.	0	.	.	.	.
Education	High school and below	−1.586	0.305	0.581	−7.212	4.040	–	–	–	–	–
	College degree	−1.572	0.990	0.320	−4.667	1.524	−0.030	0.000	0.983	−2.840	2.780
	Bachelor degree	−0.254	0.052	0.819	−2.432	1.923	0.510	0.293	0.589	−1.339	2.360
	Master degree and above	0	.	.	.	.	0	.	.	.	.
Title	Primary title	−0.351	0.040	0.841	−3.777	3.075	−1.651	0.406	0.524	−6.730	3.427
	Intermediate title	0.469	0.091	0.763	−2.581	3.519	0.532	0.050	0.824	−4.150	5.213
	Senior title	0	.	.	.	.	0.153	0.003	0.953	−4.922	5.229
	Deputy senior title	–	–	–	–	–	0	.	.	.	.
Physician classification	General practitioner	0.469	0.096	0.757	−2.501	3.439	−0.670	1.176	0.278	−1.880	0.541
	Clinician practitioner	0.079	0.002	0.968	−3.782	3.940	1.081	0.440	0.507	−2.113	4.275
	Chinese medicine practitioner	0.578	0.117	0.732	−2.731	3.888	0	.	.	.	.
	Other	0	.	.	.	.	–	–	–	–	–
Time of working (year)	Engaged in clinical work	0.041	0.812	0.368	−0.049	0.131	−0.014	0.025	0.874	−0.180	0.153
	Engaged in community health service	−0.026	0.082	0.774	−0.203	0.151	0.034	0.283	0.595	−0.093	0.161
	Working as a family doctor	−0.050	0.282	0.595	−0.234	0.134	−0.016	0.027	0.868	−0.200	0.169
	Working in community health service center	0.003	0.002	0.967	−0.147	0.153	−0.114	4.715	0.030	−0.216	−0.011
Employment forms	Officially edited	−5.803	2.543	0.111	−12.935	1.330	−3.848	3.136	0.077	−8.108	0.411
	Labor dispatch system	−7.429	3.756	0.053	−14.943	0.084	20.608	.	.	20.608	20.608
	Temporary contract system	13.116	0.000	0.999	− 14126.561	14152.794	−0.933	0.119	0.730	−6.237	4.372
	Other	0	.	.	.	.	0	.	.	.	.
Providing all the contents in the contract service package	Absolutely	6.072	4.303	0.038	0.335	11.808	−1.973	1.471	0.225	−5.160	1.215
	Basically	0.764	0.148	0.701	−3.136	4.664	−2.311	4.611	0.032	−4.421	−0.202
	Only a small part	1.085	0.270	0.603	−3.008	5.177	0	.	.	.	.
	Not at all	0	.	.	.	.	–	–	–	–	–
Ability to play and demonstrate as a family doctor	Fully play	25.589	373.111	0.000	22.993	28.185	−2.980	3.716	0.054	−6.010	0.050
	Play	25.025	744.448	0.000	23.227	26.823	−0.649	1.221	0.269	−1.800	0.502
	General	25.339	1023.207	0.000	23.786	26.891	0	.	.	.	.
	Not able to play	25.356	.	.	25.356	25.356					
	Difficult to play	0	.	.	.	.	–	–	–	–	–
Professional reputation as family doctors	Higher reputation	1.160	0.229	0.632	−3.594	5.914	5.668	3.760	0.052	−0.061	11.397
	High reputation	−0.827	0.228	0.633	−4.218	2.564	2.019	1.618	0.203	−1.092	5.131
	General reputation	1.016	1.267	0.260	−0.754	2.786	2.399	3.236	0.072	−0.215	5.013
	Low reputation	0.698	0.755	0.385	−0.876	2.272	2.066	2.531	0.112	−0.479	4.612
	Lower reputation	0	.	.	.	.	0	.	.	.	.
Social status self-evaluation (range 1–100 score)	0–50 score	−23.130	0.000	0.997	− 14162.805	14116.546	1.665	0.690	0.406	−2.265	5.595
	51–60 score	−22.974	0.000	0.997	−14162.649	14116.702	0.227	0.028	0.868	−2.445	2.898
	61–70 score	−22.747	0.000	0.997	−14162.422	14116.928	0.183	0.020	0.889	−2.384	2.750
	71–80 score	−22.482	0.000	0.998	− 14162.158	14117.193	0.361	0.064	0.801	−2.446	3.168
	81–90 score	−18.117	0.000	0.998	− 14157.793	14121.559	0	.	.	.	.
	91–100 score	0	.	.	.	.	–	–	–	–	–
Sense of work achievement (range 1–100 score)	0–50 score	5.362	5.587	0.018	0.916	9.809	−8.261	6.656	0.010	−14.536	−1.985
	51–60 score	4.662	5.766	0.016	0.857	8.467	−5.693	4.296	0.038	−11.075	−0.310
	61–70 score	4.485	5.323	0.021	0.675	8.295	−5.962	5.290	0.021	−11.042	− 0.881
	71–80 score	4.276	5.483	0.019	0.697	7.855	−6.546	6.334	0.012	−11.644	− 1.448
	81–90 score	0	.	.	.	.	−5.346	3.607	0.058	−10.863	0.171
	91–100 score	0	.	.	.	.	0	.	.	.	.
Job satisfaction	Collaborative environment satisfaction	−0.470	2.950	0.086	−1.007	0.066	0.973	7.045	0.008	0.254	1.691
	Income satisfaction	0.040	0.017	0.895	−0.553	0.633	0.042	0.019	0.889	−0.556	0.641
	Support satisfaction	0.622	4.249	0.039	0.031	1.213	−0.419	2.275	0.131	−0.963	0.125
Log pseudo likelihood		222.592					201.074				
Chi-square value (*P* value)		57.803(0.009)					37.292(0.239)				
Pseudo R^2^		0.408					0.366				

## Discussion

Chinese community health services in China are at the foundation and the Chinese medical health-care system [16]. As health-care managers for the residents in their community, family doctors are the main mechanism by which community health-care services [17–19]. The capability and level of family doctor services is central to the smooth provision of contract services, because family doctors have a good command of social medicine, preventive medicine, psychology, and other types of knowledge, as well as providing medical care services [20–23]. Therefore, the continuous improvement of family doctor service capabilities is central to the sustainable development of the family doctor responsibility system [24, 25]. After years of development, China has established a preliminary general medicine education and qualification access system, but there authoritative and scientific evaluation and assessment standards for family doctors are lacking. The absence leads to unclear goals for family doctors and uneven distribution of doctors in communities.

WONCA’s core competency model is relatively mature [26–28] and includes primary healthcare, people-centered care, problem-solving skills, comprehensive processing, community-oriented care, and overall modularization. In contrast, the development and application of a general practitioner competency model for general practitioners in China is still being debated in theoretical research [29–31]. The development and deepening of research on this competency model for general practitioner means that the model is improving constantly improving. To ensure that the model is eventually be applied to training in general medicine training, the process of optimizing general medicine training in general medicine is important [32, 33]. In 2017, Shenzhen city (Guangdong Province) issued the “Shenzhen senior family doctor competency assessment guide (trial),” which indicated a “good” direction for family doctors. Senior family doctor competency includes five aspects: i.e., professional ethics and quality; communication and interpersonal skills; general diagnosis and treatment ability; personal, family, and community health-care capacities; and the ability to use and coordinate health-related resources. The assessment guide has three advantages. First, it includes abilities other than medical ability in the assessment requirements. Second, it changes the traditional mode of “emphasizing theory over practice” in the medical and health assessment system, which means that the assessment of senior family doctors is closer to their actual work. Third, the assessment content is more consistent with actual service objects, and clearly describes how senior family doctors should provide services for specific types of service objects [34].

The degree of competency among family doctors in the Changning community health service centers is generally high. This study showed that factors affecting family doctor competency in 2013 were education background, professional title, configuration of general practitioner’s team, job training, whether the specific content of the family doctor system was clear, and the ability to demonstrate their skills as a family doctor. Moreover, family doctor competency was higher with better team configuration, while the additional job training required indicated lower competency. The greater clarity about the specific content of the family doctor system was associated with higher-qualified family doctors. The greater the family doctor’s competency, the higher their sensory ability was displayed. In 2016, job training and whether the family doctor demonstrated her/his ability were key factors affecting their work competency. In recent years, Shanghai has implemented continuous specialization in general practitioner training and general practitioner team construction has been implemented in Shanghai. Therefore, the construction and application of a competency model for general practitioners may inform the evaluation and optimization of the comprehensive quality of general practitioners and the improvement of their training system. Our study was consistent with the pilot of Shenzhen Bao’an family doctor competency assessment, which emphasized practice in family doctor training and the construction of a learning team to support training effect and ability improvement. The Weifang Community Health Service Center has set up a general practitioner training center in the Pudong New Area of Shanghai. This training center is reliant on the construction of the general practitioner theory examination question bank being developed by the Fudan University General Medicine Department, through purchases of digital simulation, hardware, and a physical examination training model to establish a network video teaching system. The training center conducts theoretical assessment for all general practitioners in Pudong. Each year, 20% of general practitioners are included in rotational operation skill training and examinations, with the purpose of promoting general practitioners’ theoretical levels and business skills and updating their knowledge. This skills training aims to update the family doctors’ skills, improve health-care service levels, and support them in becoming excellent gatekeepers for residents’ health. Personal information files for general practitioners were established and the results of practical training assessments were recorded. Furthermore, these assessment results are linked to the general practitioners’ performance subsidies [35].

In China, the service package content design, collaborative referral system, and personnel training in the family doctor system are good. However, the literature shows that family physician team stability directly affects the community’s first health-care options, two-way referral, and the formation of hierarchical diagnoses and treatment and orderly patterns of medical services. In addition, one of the major difficulties in promoting services experienced by the family doctor system is the lack of enthusiasm among family doctors. This survey found that higher competency of family doctors was related to a higher sense of their ability being displayed and demonstrated, higher sense of achievement, higher satisfaction with environment/support, and better job stability. These factors may be closely related to efforts to establish standardized construction of community health service centers in Shanghai. A good health environment and uniform medical environment with uniform equipment and consistent style can improve the patients’ experience of medical treatment. Family doctors tend to be highly satisfied with their work environment, but the mechanism of economic incentives has restricted their motivation to some extent [36]. Therefore, it is important to strengthen and improve the intrinsic value of community health services’ through the cultivation of and incentives for family doctors, and also strive for a favorable external environment for health-care reform. In April 2018, the National Health Commission of the People’s Republic of China issued a notice requiring all localities to establish assessment and evaluation mechanisms for family doctor contracting services, incorporate them into the comprehensive performance assessment of primary medical and health institutions, organize regular assessment, and link assessment results to the total performance salary of primary medical and health institutions and the salaries of the main responsible individuals. At the same time, the Medical Reform Office of the State Council promoted the “establishment of a family doctor contracting service fee system” to improve the family doctors’ work motivation as proposed in the guidance on promoting the family doctors’ contracting service. The construction of integrated rural and urban health care in Shanghai, i.e., tertiary and secondary hospitals, and community health centers, for regional community members is responsible for ensuring residents register with their community health centers, the coordinated service system construction is promoted, and a two-way referral green channel established. In this study, we found that family doctors reported relatively high satisfaction with the coordination of their neighborhood committee, medical technologies, and general practice team, but reported relatively low satisfaction with the coordination of second- and third-level hospitals. This finding suggested that it may be necessary to optimize the function of regional medical associations, effectively improve the clinical diagnosis and treatment capability and level of grass-roots medical institutions, guide patients to use their local community health-care services, and promote the construction of an effective hierarchical diagnosis and treatment system.

### Strengths and limitations

The present study has three main strengths. First, this study is a three-year follow-up study that analyzed the changes in influential factors affecting the family doctors’ work. Second, this study constructed a model of family doctors’ work competency and work stability to identify influential factors. Third, the survey of the family doctors in this study covered their work status, cognition, satisfaction, and stability, among the influential factors studied. Nevertheless, the present study has some limitations, e.g., the investigators included in the 2-year study were not all the same people in both periods; therefore, different degrees of bias may influence the results.

## Conclusions

This study found that family doctors’ satisfaction is a key factor affecting their work stability. Family doctors’ work competency can affect their job stability, and their work cognition may be important for work competency and stability.

## Data Availability

The data generated and analyzed during the present study are not publicly available because of data sensitiveness. However, they are available from the Academic Ethics Committee of Shanghai Pudong Institute for Health Development (via yanhua373@126.com) for researchers who meet the criteria for access to confidential data.

## Abbreviation

WONCA: World Organization of National Colleges, Academies and Academic Association of General Practitioners/Family Physicians
